# Supplementary material for: Environmental Factors Affecting Feather Taphonomy
Source: Biology (Basel). 2022 May 3;11(5):703. doi: 10.3390/biology11050703 (PMC9138376; doi:10.3390/biology11050703)
Supplement: Supplementary file 1 [file biology-11-00703-s001.zip › biology-1636089-supplementary.pdf]

Environmental Factors Affecting Feather Taphonomy

Supplemental Materials

Materials and Methods

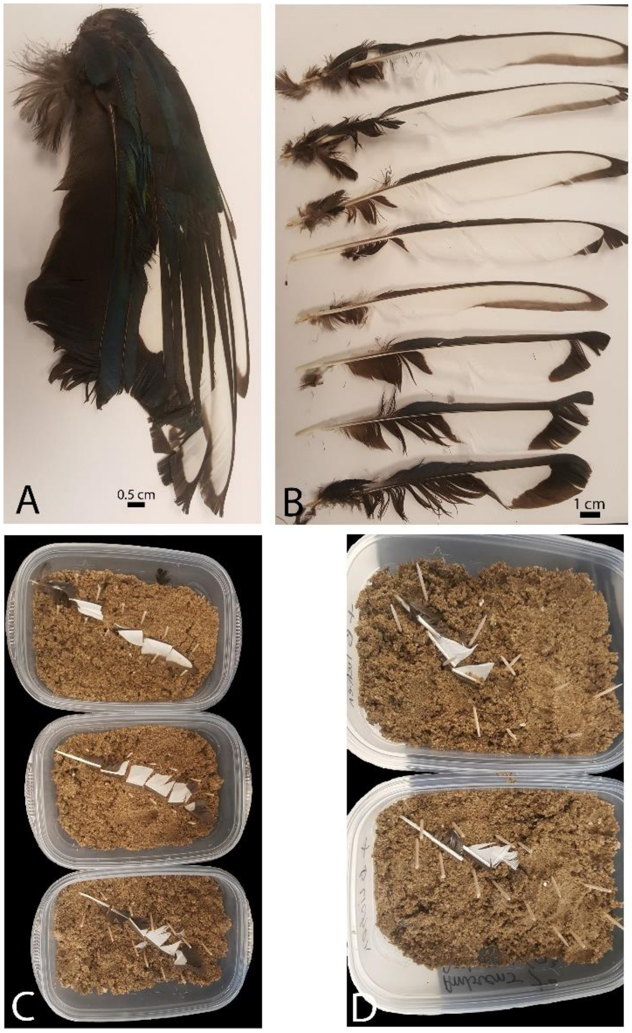

Figure S1. Experimental setup. All conditions were identical in distribution. (A) Entire wing of *Pica hudsonia*, showing the distribution of pigmented feathers. (B) Examples of individual feathers used in this experiment. (C) Feathers placed on top of sediments, either sand or natural pond sediments. (D) Feathers were marked with toothpicks for ease of recovery after degradation; in each setup, half of the feather was exposed (left) and half was buried (right).

Black and white magpie feathers (*Pica hudsonia*) were cut into pieces approximately 2 cm in length and placed in containers measuring 8 x 6 x 2.5 in. For all conditions, sediments (either pond sediments collected from the bottom of a shallow, local North Carolina pond in which many different bird species reside, or commercial sand) were placed at the bottom of the container in a 3 cm layer, and the container was filled with pondwater 0.5 cm above the sediment. Half of the feather was buried in the middle layer of the sediment, and some was left unburied above the sediment, as shown in Figure S1. The containers were loosely covered with lids. Each of these was prepared in duplicate, with one exposed to ambient atmosphere and one to elevated CO^2 atmospheres. The feathers were then exposed to the following conditions, as described in the text and in Table 1.

A) **Pond (Sediment and Pondwater) (Buried and Unburied)**

Pond sediment was placed at the bottom of the container in a 3 cm layer, and the container was filled with pondwater 0.5 cm above the sediment. Half of the feather was buried in the middle layer of the sediment, and some was left unburied above the sediment, as shown in Figure S1D. The containers were loosely covered with lids.

B). **Pond (Sediment and Pondwater) + *B.licheniformis* (Buried and Unburied)**

Pond sediment was placed at the bottom of the container in a 3 cm layer, and the container was filled with pondwater 0.5 cm above the sediment. There was 1.1 in of empty space above the water. Feathers were soaked in pure cultures of *Bacillus licheniformis*, and then some were buried in the middle layer of the sediment and some left unburied above the sediment, as described above.

C) **Pond (Sediment and Pondwater) + Calcium Hydroxyapatite (Buried and Unburied)**

Pond sediment was distributed as above, and the container was filled with a calcium phosphate solution that consisted of 0.1 M $CaCl_2$ and 0.1 M NaH_2PO_4 dissolved in pH 5 pondwater. This solution was prepared as follows: 180 ml 0.1 M NaH_2PO_4 in pondwater was slowly added to 300 ml 0.1 M $CaCl_2$ in pondwater (final pH 4.8) [1] to cover the sediment to 0.5 cm, as above. There was 1.1 in of empty space above the water. Half of the feather was buried in the middle layer of the sediment, and half was left unburied above the sediment. The container was covered with a loose lid.

D) **Sand (sand and E-Pure Water) + Calcium Hydroxyapatite (Buried and Unburied)**

QuikRETE premium 1113 sand was placed at the bottom of the container, as above. The container was filled with a calcium phosphate solution as described above, and feathers were either buried or left on the sediment surface, as described.

E) **Sand (Sand and Pure Water) (Buried and Unburied)**

QuikRETE premium 1113 sand was placed at the bottom of the container and pure deionized or DI water was added to 0.5 cm above the sediment.

Toothpicks were used to mark the burial position to retrieve the feather samples in all cases (Figure S1D). Duplicate samples were prepared using separate loosely capped containers. One was placed in a 30 $^{\circ}$C incubator (Forma Scientific Inc.) in 0.5% CO_2 without an external light source, and another one was left at ambient pressure and room temperature (~24 $^{\circ}$C under GE cool light in daytime, dark at night). The liquid level in each container was kept constant by refilling the corresponding liquids (e.g., pond water, calcium phosphate solution, or pure DI water) periodically. Small pieces of each feather were collected for analysis at intervals of two weeks.

All collected feather samples after degradation were gently transferred to a Biopsy Uni-Cassette (Tissue-Tek Cat #4088), immersed in 10% neutralized formalin and fixed for 1 h at RT, and then rinsed with PBS and pure water and air dried in the Biosafety Hood overnight for LM, SEM imaging, and LR white embedding.

SEM

The air-dried samples were mounted on aluminum stubs with double-sided carbon tape. Stubs were placed in a sputter coater, where ~10 nm of iridium was deposited to make the surface conductive for SEM imaging. Images were taken at a working distance of 4 mm and at 1.00 kV.

TEM

Air-dried feathers were embedded in LR White resin blocks after partial dehydration in 70% ethanol and thorough infiltration with concentrated LR White. The specimen and embedding medium were placed in a gelatin capsule (EMS Cat #70100) and allowed to polymerize for 48 h at 60 °C. Sections of a total of 90 nm were taken on a Leica EM UC6 Ultramicrotome, mounted on carbon-coated nickel grids (EMS Cat CFT200-NI), and stained with 5% methanolic uranyl acetate for 5 min and Reynold's lead citrate for 8 min, and then observed using the FEI Talos F200X G2 electron microscope in the AIF at North Carolina State University at 200kv S/TEM (scanning/transmission electron microscopes) with both a bright field detector (BF) and a high-angle annular dark field detector (HAADF).

In Situ Immunohistochemistry (IHC)

Sections of a total of 200 nm were taken on a Leica EM UC6 Ultramicrotome and dried overnight at 45 °C to each well of a PTFE-printed slide (EMS Cat#63424-06). All sections were etched with 25 ug/ml proteinase K and 0.5 MEDTA for epitope retrieval, followed by 1 mg/ml sodium borohydride incubation for quenching autofluorescence. Unspecific binding was blocked with 4% goat normal serum, and then sections were immunolabelled with primary antibodies (rabbit anti-feather (Biosynthesis BYSN6733)) in a ratio of 1:200 in primary dilution buffer (1% bovine serum albumin (BSA) (Fisher, BP1660-100), 0.1% cold fish skin gelatin (Sigma G7765), 0.05% sodium azide (Sigma S-8032), 0.01M PBS (pH 7.2)) or in primary dilution buffer only, without primary antibody added, to control for non-specific secondary antibody binding. All sections were washed thoroughly to remove unbound antibody, then incubated with secondary antibody (biotinylated goat anti-rabbit IgG (H+L) (Vector BA-1000)) diluted in a ratio of 1:500 for 2 h at room temperature. Fluorescein Avidin D (Vector Laboratories A-2001) was applied for 1 h. All incubations were separated by sequential washes. Finally, the sections were mounted with Anti-Fade mounting medium (Vector H-1000) and coverslips were applied. The sections were examined with a Zeiss Axioskop 2 Plus biological microscope. The original transmitted light images were adjusted to increase contrast by 68. FITC images were not affected and are as captured.

ACKNOWLEDGMENTS

This work was performed in part at the Analytical Instrumentation Facility (AIF) at North Carolina State University, which is supported by the State of North Carolina and the National Science Foundation **(award number ECCS-2025064)**. The AIF is a member of the North Carolina Research Triangle Nanotechnology Network (RTNN), a site in the National Nanotechnology Coordinated Infrastructure (NNCI).
